# Telemedicine-Supported CPAP Therapy in Patients with Obstructive Sleep Apnea: Association with Treatment Adherence and Clinical Outcomes

**DOI:** 10.3390/jcm14155339

**Published:** 2025-07-29

**Authors:** Norbert Wellmann, Versavia Maria Ancusa, Monica Steluta Marc, Ana Adriana Trusculescu, Camelia Corina Pescaru, Flavia Gabriela Martis, Ioana Ciortea, Alexandru Florian Crisan, Adelina Maritescu, Madalina Alexandra Balica, Ovidiu Fira-Mladinescu

**Affiliations:** 1 Doctoral School, “Victor Babes” University of Medicine and Pharmacy Timisoara, Eftimie Murgu Square 2, 300041 Timisoara, Romania; norbert.wellmann@umft.ro (N.W.); flavia.martis@umft.ro (F.G.M.); ioana.ciortea@umft.ro (I.C.); adelina.maritescu@umft.ro (A.M.); madalina.balica@umft.ro (M.A.B.); 2 Center for Research and Innovation in Personalized Medicine of Respiratory Diseases (CRIPMRD), “Victor Babes”, Pneumology University Clinic, University of Medicine and Pharmacy Timisoara, Eftimie Murgu Square 2, 300041 Timisoara, Romania; pescaru.camelia@umft.ro (C.C.P.); mladinescu@umft.ro (O.F.-M.); 3 Pulmonology Department II, Clinical Hospital of Infectious Diseases and Pneumophthisiology, “Dr. Victor Babes” Timisoara, Gheorghe Adam Street, No 13, 300310 Timisoara, Romania; 4 Department of Computer and Information Technology, Automation and Computers Faculty, “Politehnica” University of Timisoara, Vasile Pârvan Blvd, No. 2, 300223 Timisoara, Romania; versavia.ancusa@upt.ro; 5 Pulmonary Rehabilitation Center, Clinical Hospital of Infectious Diseases and Pulmonology, “Dr. Victor Babes” Timisoara, Gheorghe Adam Street, No. 13, 300310 Timisoara, Romania; crisan@umft.ro; 6 Research Center for the Assessment of Human Motion, Functionality and Disability (CEMFD), “Victor Babes” University of Medicine and Pharmacy Timisoara, Eftimie Murgu Square 2, 300041 Timisoara, Romania; 7 Infectious Diseases University Clinic, Department of Infectious Diseases, “Victor Babes” University of Medicine and Pharmacy Timisoara, Eftimie Murgu Square 2, 300041 Timisoara, Romania

**Keywords:** obstructive sleep apnea, telemedicine, continuous positive airway pressure, remote monitoring, patient adherence, AirView™, machine learning, cluster analysis

## Abstract

**Background/Objectives:** Obstructive sleep apnea (OSA) is a highly prevalent disorder that significantly impacts quality of life and daily functioning. While continuous positive airway pressure (CPAP) therapy is effective, long-term adherence remains a challenge. This single-arm observational study aimed to evaluate clinical outcomes and adherence patterns during telemedicine-supported CPAP therapy and identify distinct phenotypic response clusters in Romanian patients with OSA. **Methods:** This prospective observational study included 86 adults diagnosed with OSA, treated with ResMed Auto CPAP devices at “Victor Babeș” University Hospital in Timișoara, Romania. All patients were remotely monitored via the AirView™ platform and received monthly telephone interventions to promote adherence when necessary. Clinical outcomes were assessed through objective telemonitoring data. K-means clustering and t-distributed stochastic neighbor embedding (t-SNE) were employed to explore phenotypic response patterns. **Results:** During telemedicine-supported CPAP therapy, significant clinical improvements were observed. The apnea–hypopnea index (AHI) decreased from 42.0 ± 21.1 to 1.9 ± 1.3 events/hour. CPAP adherence improved from 75.5% to 90.5% over six months. Average daily usage increased from 348.4 ± 85.8 to 384.2 ± 65.2 min. However, post hoc analysis revealed significant concerns about the validity of self-reported psychological improvements. Self-esteem changes showed negligible correlation with objective clinical measures (r < 0.2, all *p* > 0.1), with only 3.3% of variance being explained by measurable therapeutic factors (R^2^ = 0.033). Clustering analysis identified four distinct adherence and outcome profiles, yet paradoxically, patients with lower adherence showed greater self-esteem improvements, contradicting therapeutic causation. **Conclusions:** Telemedicine-supported CPAP therapy with structured monthly interventions was associated with substantial clinical improvements, including excellent AHI reduction (22-fold) and high adherence rates (+15% after 6 months). Data-driven phenotyping successfully identified distinct patient response profiles, supporting personalized management approaches. However, the single-arm design prevents definitive attribution of improvements to telemonitoring versus natural adaptation or placebo effects. Self-reported psychological outcomes showed concerning patterns suggesting predominant placebo responses rather than therapeutic benefits. While the overall findings demonstrate the potential value of structured telemonitoring for objective CPAP outcomes, controlled trials are essential to establishing true therapeutic efficacy and distinguishing intervention effects from measurement bias.

## 1. Introduction

Sleep-related breathing disorders (SRBDs), including obstructive sleep apnea (OSA), are characterized by recurrent interruptions in breathing during sleep due to upper-airway obstruction. These events result in fragmented sleep and intermittent hypoxemia, leading to symptoms such as excessive daytime sleepiness, fatigue, mood disturbances, and cognitive impairment. OSA is a highly prevalent condition, affecting 14% to 49% of middle-aged men in the United States and Europe, and is associated with significant cardiovascular and metabolic risks. The International Classification of Sleep Disorders (ICSD-3) categorizes OSA among the primary sleep disorders and recognizes its growing impact on public health [1,2,3,4,5,6,7,8,9].

Telemedicine has emerged as an effective tool in the management of chronic diseases such as OSA, enabling remote monitoring, diagnosis, education, and follow-up through digital platforms. In particular, CPAP (continuous positive airway pressure) therapy, the gold standard treatment for OSA, benefits from telemonitoring technologies such as the AirView™ system. These systems automatically transmit data on CPAP adherence, residual apnea–hypopnea index (AHI), leak rates, and pressure settings to healthcare providers. Moreover, telemedicine has been shown to improve patient engagement, adherence to therapy, and clinical outcomes while also reducing the burden on healthcare systems and shortening waiting times for consultations [10,11,12,13,14,15,16,17,18,19].

To assess patient outcomes, validated tools such as the STOP-BANG Questionnaire and the Epworth Sleepiness Scale are commonly used to screen for OSA and evaluate the levels of daytime sleepiness, respectively. Quality of life is often measured using the WHOQOL-BREF questionnaire, a shortened version developed by the World Health Organization. It includes 26 items across four domains: physical health, psychological health, social relationships, and environmental well-being. The Romanian version of the WHOQOL-BREF is publicly available on the WHO website. Additionally, psychological status is evaluated using the Rosenberg Self-Esteem Scale, which has also been validated in Romanian. These instruments allow for the comprehensive evaluation of the physical and psychosocial impact of OSA and the effectiveness of CPAP treatment [20,21,22,23,24,25,26,27,28,29,30].

While CPAP therapy is a cornerstone for obstructive sleep apnea, a comprehensive understanding of its impact on holistic patient well-being (beyond just clinical outcomes) and of the inherent heterogeneity of patient responses—particularly within the context of evolving telemedicine-based follow-up models—remains an underexplored area. Addressing this gap, this study aimed to evaluate the clinical, psychological, and quality-of-life outcomes of patients undergoing CPAP therapy with telemedicine-based follow-up via the AirView™ platform. Furthermore, to better characterize individualized treatment benefits and inform personalized care, unsupervised machine learning was applied to identify distinct response-based phenotypic clusters over a six-month period.

## 2. Materials and Methods

### 2.1. Study Design and Setting

This prospective observational study enrolled 86 adult patients diagnosed with obstructive sleep apnea (OSA) who initiated treatment with ResMed Auto CPAP devices (AirSense™ 10 and AirSense™ 11 AutoSet, ResMed, San Diego, CA, USA) and were followed over a six-month period. The study was conducted at “Victor Babeș” University Hospital in Timișoara, Romania, between October 2024 and June 2025.

### 2.2. Ethical Approval

This study received ethical approval from both the university ethics committee (approval No. 49/01.10.2024) and the hospital ethics committee (approval No. 8528/25.09.2024). All participants provided written informed consent prior to inclusion.

### 2.3. Participants

Adult patients aged 18 years or older with confirmed moderate to severe OSA were eligible for inclusion. The study population comprised patients with substantial sleep-disordered breathing (mean AHI of 42.0 ± 21.1 events/hour; 65.1% with severe OSA) and significant comorbidity burden (mean BMI of 35.3 kg/m^2^, with 79.1% of patients being obese), representing a challenging cohort with multiple established risk factors for poor CPAP adherence.

The intensive telemonitoring intervention was clinically justified by the high-risk population characteristics, including advanced age (mean of 60.5 years), severe obesity predominance, 100% treatment-naive status, and marked baseline functional impairment across multiple domains. This population profile aligned with established predictors of CPAP non-adherence, supporting the rationale for structured remote monitoring and targeted adherence interventions.

Additional inclusion criteria were access to a mobile phone or landline for telephonic follow-up, ability to use CPAP equipment independently or with assistance, willingness to participate in monthly telemonitoring and phone-based interventions, and initiation of treatment with ResMed Auto CPAP devices. Exclusion criteria included age under 18 years, diagnosis of central or mixed sleep apnea, severe psychiatric or cognitive impairment that could interfere with treatment adherence or completion of study questionnaires, inability to communicate by phone, lack of internet or electricity access required for CPAP telemonitoring, and refusal to participate.

### 2.4. Data Collection and Management

Data were systematically collected and organized using Microsoft Excel, including demographic, clinical, respiratory, and psychosocial variables assessed at baseline and throughout the six-month follow-up period. To ensure data integrity and patient confidentiality, all records were anonymized by assigning unique patient identifiers (P001-P086).

### 2.5. Remote Monitoring and Adherence Support

#### Rationale for Human-Delivered Phone Interventions

**Figure 1:**
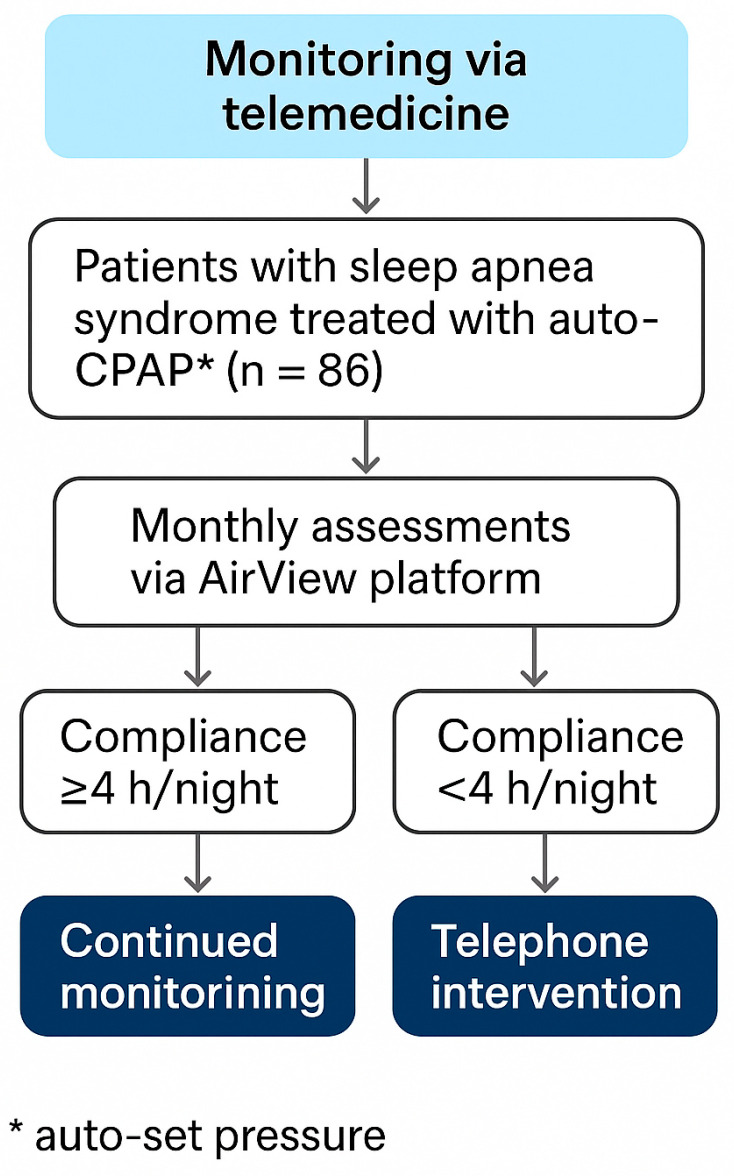
The monitoring was structured according to a compliance check.

All patients were remotely monitored (Figure 1) using the AirView™ telemedicine platform (ResMed), which continuously transmitted key therapy data to the clinical team. The platform provided real-time information on CPAP adherence (minutes of use per night), residual apnea–hypopnea index (AHI), leak rates, and pressure parameters, enabling comprehensive and ongoing assessment of treatment effectiveness and device performance throughout the study period.

Monthly telephone interventions were deliberately conducted by trained healthcare professionals rather than automated or AI-assisted systems. This decision was based on the psychological profile of our patient population, which included significant baseline impairments in self-esteem (79% below normal range) and quality of life across all domains. Patients with OSA frequently present with concurrent depression, anxiety, and social isolation that require careful human assessment and empathetic support during treatment adaptation.

The use of human contact was considered essential to patient safety, particularly given emerging evidence of potential risks associated with AI interactions in psychologically vulnerable populations. The healthcare professionals conducting the calls were trained to assess not only CPAP adherence and technical issues but also psychological well-being, treatment-related distress, and the need for additional clinical support. This approach ensured that patients received genuine empathy, clinical judgment, and appropriate escalation of concerns that current AI systems cannot reliably provide.

The monthly calls served a dual purpose: objective adherence monitoring through AirView™ data review and subjective assessment of patient adaptation, concerns, and psychological status. This human-centered approach was deemed crucial to maintaining the therapeutic relationship and ensuring comprehensive patient care during the vulnerable period of CPAP therapy initiation.

Telephone interventions once a month were conducted for patients demonstrating suboptimal adherence (defined as average usage of less than 4 h per night based on AirView™ data). Each intervention followed a standardized protocol administered by trained healthcare professionals and typically lasted 15–20 min. The structured intervention comprised three core components: technical support and device optimization, educational counseling and motivation enhancement, and sleep hygiene optimization.

The technical support component involved a systematic assessment of device-related issues through guided troubleshooting. Personalized mask fitting guidance was provided, including alternative mask recommendations when indicated. Comfort-related problems such as pressure intolerance, claustrophobia, and air leaks were addressed individually. Patients also received education on equipment maintenance, including cleaning protocols and replacement schedules. Where appropriate, clinicians reviewed AirView™ data to offer pressure setting optimization tailored to the patient’s specific therapy profile.

Educational counseling focused on reinforcing the benefits of CPAP therapy, using personalized data such as AHI reduction trends from AirView™ reports. Patients were educated about the health consequences of untreated OSA, with attention to their individual cardiovascular, metabolic, and cognitive risk factors. The importance of long-term adherence was emphasized, and motivational interviewing techniques were used to address ambivalence. Each session included collaborative goal setting aimed at incremental improvements in usage, with realistic and achievable milestones.

The sleep hygiene optimization segment assessed the patient’s current sleep environment and bedtime routines. Based on individual lifestyle factors, personalized recommendations were offered, including guidance on optimal sleep scheduling and maintaining circadian rhythm stability. Environmental modifications were suggested to improve sleep quality and CPAP comfort, such as adjustments to noise, light, or temperature. Additionally, stress management strategies were discussed to support better sleep initiation and maintenance, enhancing overall therapy tolerance.

### 2.6. Clinical Assessments

**Quality of Life**: Assessed using the short version of the WHOQOL-BREF questionnaire in the official Romanian language version available on the WHO website. This instrument evaluates four domains: physical health, psychological health, social relationships, and environmental health. Scores were calculated using the standard WHOQOL-BREF formula, with domain scores transformed onto a 0–100 scale.

**Self-Esteem**: Measured using the Romanian version of the Rosenberg Self-Esteem Scale at baseline and after six months to assess psychological well-being. Self-esteem was assessed using the Rosenberg Self-Esteem Scale (RSE), scored on a 1–4 Likert scale (1 = Strongly agree; 4 = Strongly disagree), based on the original guidelines provided in the Rosenberg Self-Esteem Scale – Measures Package (2006). Scores were not converted to a 0–3 scale, yielding a total range of 10–40, with higher scores indicating greater self-esteem [30].

**Data Preprocessing for Machine Learning Analysis**

**Database Preparation**

The clinical dataset required extensive preprocessing to ensure compatibility with unsupervised machine learning algorithms, including the steps below.

Data Anonymization: Patient identifiers were replaced with sequential anonymous codes (P001-P086).

Column Header Standardization: Multi-row column headers were consolidated into single-row format for computational processing.

Language Standardization: All variable names were translated from Romanian into English for analytical consistency.

Variable Encoding: Categorical variables were numerically encoded (e.g., sex: M/F → 0/1) to meet algorithm requirements.

**Correlation Analysis**

To assess multicollinearity among variables, Pearson correlation coefficients were calculated for all numeric variables. A correlation matrix was generated and visualized as a heatmap to identify potentially redundant variables (correlation coefficients >0.7 or <−0.7). Results were exported both in CSV format and as high-resolution visualization for comprehensive review.

### 2.7. Unsupervised Machine Learning Analysis

#### 2.7.1. Optimal Cluster Number Determination

Two complementary statistical methods were employed to determine the optimal number of clusters for patient phenotyping.

**Elbow Method**: K-means clustering was performed for k values ranging from 1 to 10. The within-cluster sum of squares (WCSS) was plotted against the number of clusters to identify the “elbow point”, where the rate of WCSS decrease begins to plateau, indicating diminishing returns for additional clusters.

**Gap Statistic Method**: This method compares the within-cluster dispersion of the actual patient data to that of a reference null distribution (uniformly random data). The optimal k is selected as the smallest value satisfying Gap(k) ≥ Gap(k + 1) − s(k + 1), where s(k + 1) represents the standard error, providing a statistically robust criterion for cluster selection.

#### 2.7.2. K-Means Clustering Implementation

Based on optimization results indicating potential optimal values of k = 4, 5, and 6, k-means clustering was performed for all three scenarios to enable comparative analysis. The scikit-learn implementation was utilized with the following parameters: random state fixed for reproducibility, maximum iterations set to 300, and initialization using k-means++ for improved convergence.

#### 2.7.3. Dimensionality Reduction and Visualization

To visualize high-dimensional patient clustering results, t-distributed stochastic neighbor embedding (t-SNE) was applied to reduce the multi-dimensional feature space to two dimensions for interpretable visualization. t-SNE parameters were optimized as follows:

- Perplexity: 30.
- Learning rate: 200.
- Maximum iterations: 1000.

Clustering results were visualized as scatter plots with distinct colors representing different patient phenotypes.

#### 2.7.4. Cluster Quality Assessment

Three established internal validation metrics were calculated to quantitatively assess clustering quality and guide optimal cluster selection:

1. **Silhouette Score**: Measure of cohesion within clusters relative to separation between clusters (range of −1 to 1, where higher indicates better clustering). We obtained a maximum value of 0.21 at k = 4.
2. **Calinski–Harabasz Index**: Ratio of between-cluster to within-cluster dispersion (higher values indicate better-defined clusters). This index had a maximum of 33.60 at k = 4.
3. **Davies–Bouldin Score**: Average similarity ratio of each cluster with its most similar cluster (lower values indicate better clustering). This score also indicated k = 4 as optimal with a low value of 1.40.

#### 2.7.5. Feature Categorization

For clinical interpretability, patient variables were systematically grouped into four clinically relevant categories:

- Demographics: Age and Body Mass Index (BMI).
- Baseline Diagnosis: Initial AHI, minimum oxygen saturation, and Epworth Sleepiness Scale score.
- Treatment Adherence: Compliance > 4 h/night at 6 months and average nightly usage (minutes) at 6 months.
- Clinical Outcomes: AHI at 6 months and WHOQOL-BREF average score at 6 months.

#### 2.7.6. Statistical Analysis and Reporting

All statistical analyses were conducted using Python (version 3.11.12) within a validated computational environment. Data manipulation and preprocessing were executed using the NumPy (2.0.2) and Pandas (2.2.2) libraries to ensure reproducible data transformation workflows. Statistical inference was performed via SciPy (1.15.3) and StatsModels (0.14.4), employing appropriate parametric and non-parametric methodologies based on data distribution characteristics.

The k-means clustering algorithm and associated validation metrics were implemented through the Scikit-learn package (1.6.1), with hyperparameter optimization conducted via cross-validation procedures. Visualization of multi-dimensional data and resultant clusters was performed through Matplotlib (3.10.0) and Seaborn (0.13.2) libraries, employing standardized palettes for consistent interpretation across graphical outputs. Patient phenotype characteristics were summarized using descriptive statistics and visualized using radar plots for multi-dimensional comparison across identified clusters.

This prospective observational study enrolled 86 adult patients diagnosed with obstructive sleep apnea (OSA) who initiated treatment with ResMed Auto CPAP devices and were followed over a six-month period.

## 3. Results

### 3.1. Comprehensive Baseline Population Characterization

The study cohort comprised 86 patients with moderate to severe obstructive sleep apnea, representing a challenging population with multiple risk factors for poor CPAP adherence.

**Demographics and Anthropometric Profile:** The cohort was predominantly composed of males (72.1%, *n* = 62) of advanced age (mean of 60.5 ± 11.9 years; range: 34–89). A substantial obesity burden was present, with a mean BMI of 35.3 ± 8.0 kg/m^2^ and 79.1% of patients being classified as obese (BMI ≥ 30). The obesity distribution included Class I obesity in 27 patients (31.4%), Class II in 19 patients (22.1%), and Class III (severe obesity) in 22 patients (25.6%) (Table 1).

### 3.2. Treatment Efficacy: Respiratory Parameters

OSA Severity and Sleep-Disordered Breathing: The population demonstrated substantial sleep-disordered breathing with a mean AHI of 42.0 ± 21.1 events/hour (range: 16.8–104.5). Severe OSA (AHI ≥ 30) predominated, affecting 56 patients (65.1%), with a mean AHI of 52.1 ± 22.4 events/hour in this subgroup. Moderate OSA (AHI of 15–29.9) was present in 30 patients (34.9%) with a mean AHI of 23.1 ± 4.0 events/hour. Significant nocturnal hypoxemia was evident, with a mean desaturation index of 41.3 ± 22.0 events/hour and minimum oxygen saturation of 70.8 ± 12.0% (range: 38–87%) (Table 2).

### 3.3. Compliance Rates

Compliance with CPAP therapy, defined as usage of more than 4 h per night, showed progressive improvement throughout the study period. After 7 days, compliance was 75.5 ± 23.9%, increasing to 81.2 ± 17.4% at 1 month, and reaching 90.5 ± 10.1% by 6 months (Table 3).

### 3.4. Daily Usage

Average daily CPAP usage increased steadily from 348.4 ± 85.8 min in the first month to 384.2 ± 65.2 min at six months (Table 4).

#### Impact of Telemonitoring Support

Non-compliant patients received regular monthly telephone support, which resulted in progressively higher average nightly usage and improved compliance percentages across time points.

### 3.5. Self-Reported Outcome Measures: Quality of Life and Psychological Outcomes

#### 3.5.1. Self-Esteem Assessment and Validation Analysis

Rosenberg Self-Esteem Scale scores demonstrated substantial numerical changes from baseline (20.1 ± 5.9) to six months (30.2 ± 5.4), representing a very large effect size (Cohen’s d = 1.78) that warrants careful methodological scrutiny (Table 5).

**Figure 2:**
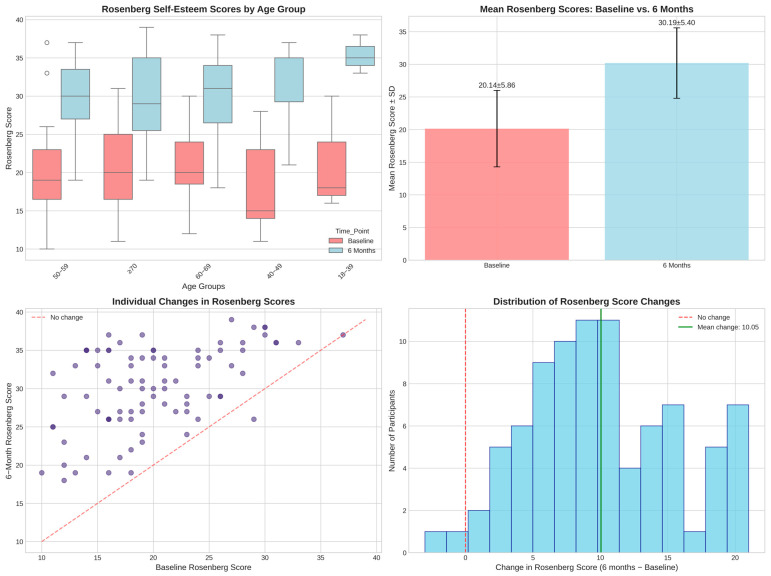
Rosenberg Self-Esteem scores: baseline vs. 6 months.

Individual patient progression is illustrated in Figure 2.

Correlation with Objective Clinical Measures: Post hoc validation analysis revealed weak correlations between self-esteem improvements and all objective treatment parameters:

Self-esteem changes vs. AHI reduction: r = 0.164 (*p* = 0.132).
Self-esteem changes vs. CPAP compliance: r = −0.081 (*p* = 0.460).
Self-esteem changes vs. daily usage minutes: r = −0.051 (*p* = 0.642).
Self-esteem changes vs. WHOQOL improvement: r = 0.153 (*p* = 0.159).

All correlations fell below the threshold for meaningful relationships (r < 0.3), indicating that self-esteem changes occurred largely independently of measurable treatment effectiveness.

Dose–Response Analysis: The examination of self-esteem improvement by treatment adherence revealed a concerning inverse pattern:

Low compliance (<70%, n = 2): 13.0 ± 7.1-point improvement.
Medium compliance (70–89%, n = 30): 10.0 ± 5.5-point improvement.
High compliance (≥90%, n = 54): 10.0 ± 5.7-point improvement.

This inverse dose–response relationship contradicts expected therapeutic mechanisms and suggests non-clinical factors driving the observed changes.

Variance Explanation Analysis: Multiple regression analysis incorporating AHI reduction, compliance, and usage patterns explained only 3.3% of the variance in self-esteem improvements (R^2^ = 0.033). This indicates that 96.7% of the observed psychological changes cannot be attributed to measurable clinical factors, strongly suggesting placebo effects, response bias, or measurement artifacts.

#### 3.5.2. Cluster-Based Validation

Our phenotypic clustering analysis provided additional evidence questioning the therapeutic nature of self-esteem improvements. Despite achieving similarly excellent AHI reductions across all clusters (92.5–95.9%), self-esteem changes varied paradoxically:

Cluster 1 (best clinical outcomes: 95.9% AHI reduction): 9.8 ± 5.9-point SE improvement.
Cluster 0 (94.1% AHI reduction): 9.1 ± 6.2-point SE improvement.
Cluster 2 (94.8% AHI reduction): 8.9 ± 6.9-point SE improvement.
Cluster 3 (worst clinical outcomes: 92.5% AHI reduction): 11.1 ± 4.5-point SE improvement.

The negative correlation between cluster-level clinical effectiveness and self-esteem improvement (r = −0.609) contradicts therapeutic causation and reinforces concerns about measurement validity.

#### 3.5.3. Quality-of-Life Measurements

**Figure 3:**
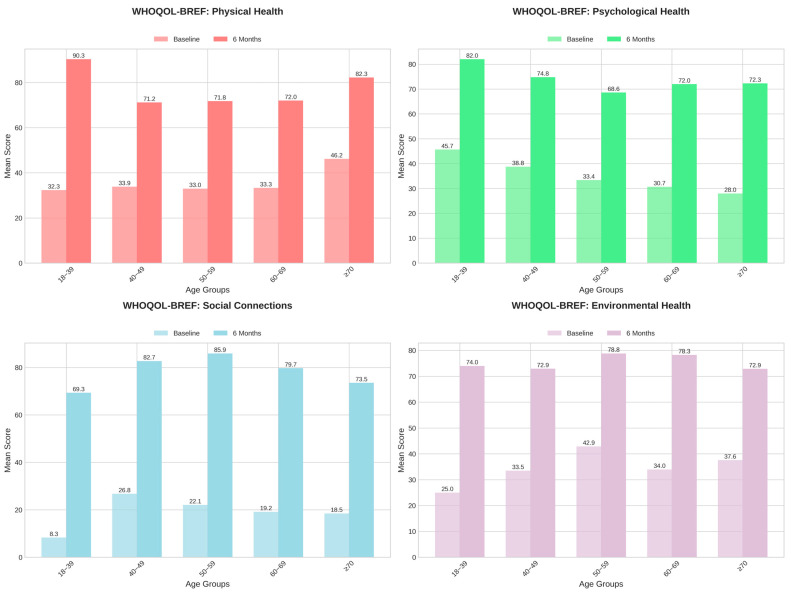

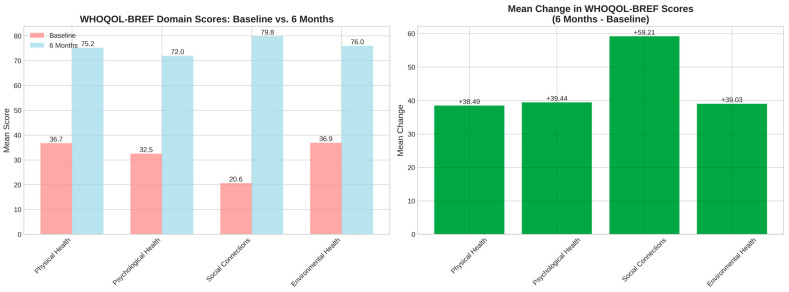
WHOQOL-BREF scores: baseline vs. 6 months.

Similar patterns were observed in WHOQOL-BREF domain scores, with substantial numerical improvements across all four domains, as detailed in Table 5 and visualized in Figure 3. From baseline to six months, physical health increased from 36.7 ± 25.4 to 75.2 ± 21.2, psychological health improved from 32.5 ± 27.0 to 71.9 ± 24.1, social relationships rose from 20.6 ± 19.1 to 79.8 ± 16.5, and environmental health increased from 36.9 ± 25.4 to 75.9 ± 21.9. The largest gains were observed in the social relationships and physical health domains, suggesting meaningful subjective benefit. While these improvements may reflect genuine patient experiences, the lack of a controlled comparison limits the ability to attribute these changes solely to the therapeutic intervention, as expectation or placebo effects cannot be excluded.

#### 3.5.4. Methodological Implications

These findings highlight critical limitations inherent in single-arm studies for evaluating subjective psychological outcomes and demonstrate the necessity of controlled designs to distinguish genuine therapeutic benefits from well-documented biases, including

- Hawthorne effects from intensive monitoring.
- Social desirability bias in patient–provider interactions.
- Response shift bias following treatment initiation.
- Placebo responses to perceived “high-tech” interventions.

### 3.6. Patient Phenotyping and Clustering Analysis

#### 3.6.1. Demographic Patterns

**Figure 4:**
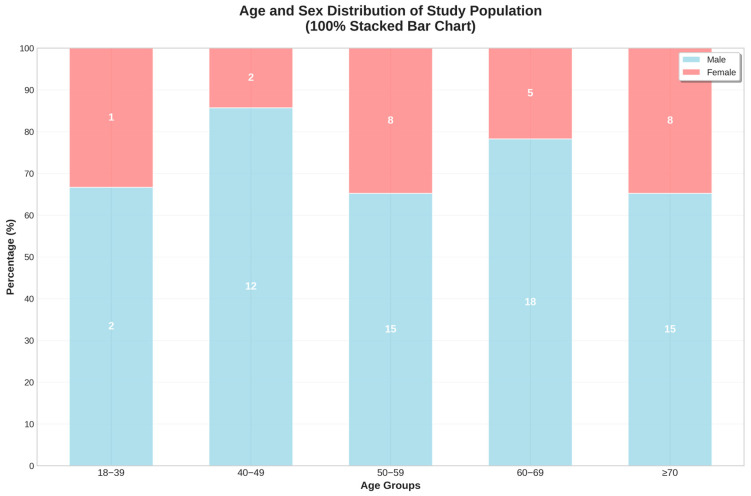
Age and sex distributions of the study population.

Age and sex distribution analyses confirmed male predominance across all age groups, with the highest concentrations in the 40–49 and 60–69 age brackets (Figure 4).

**Figure 5:**
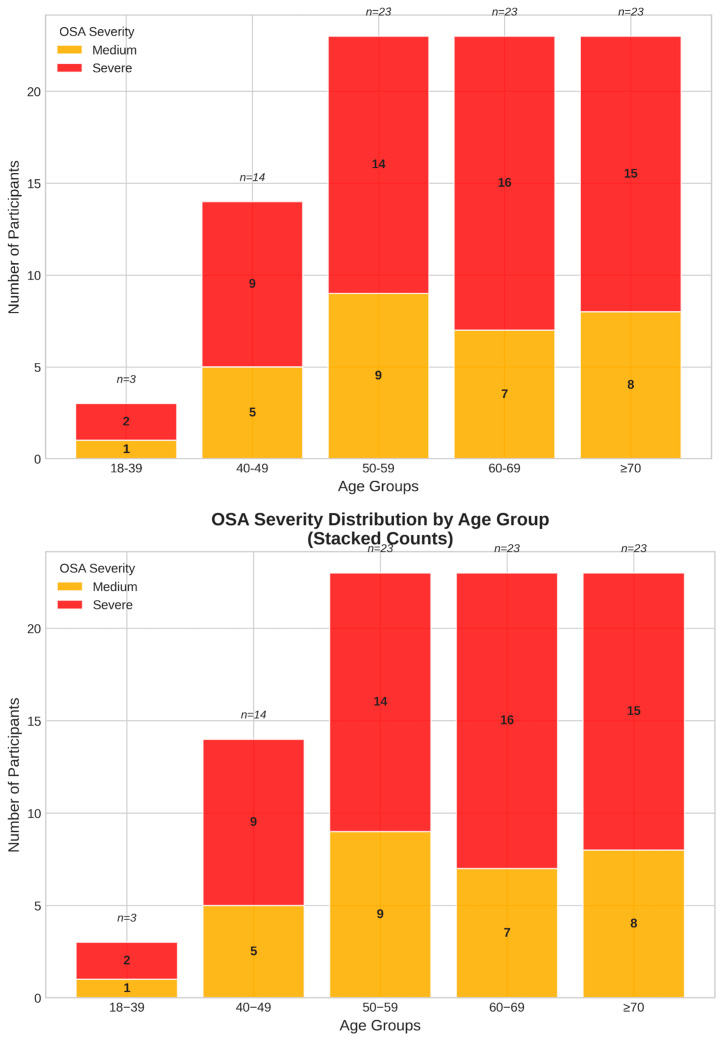
Severity of OSA by age group.

Severe OSA was more prevalent in older age groups, particularly in patients aged 60–69 and ≥70 years (Figure 5).

#### 3.6.2. Machine Learning Clustering

**Figure 6:**
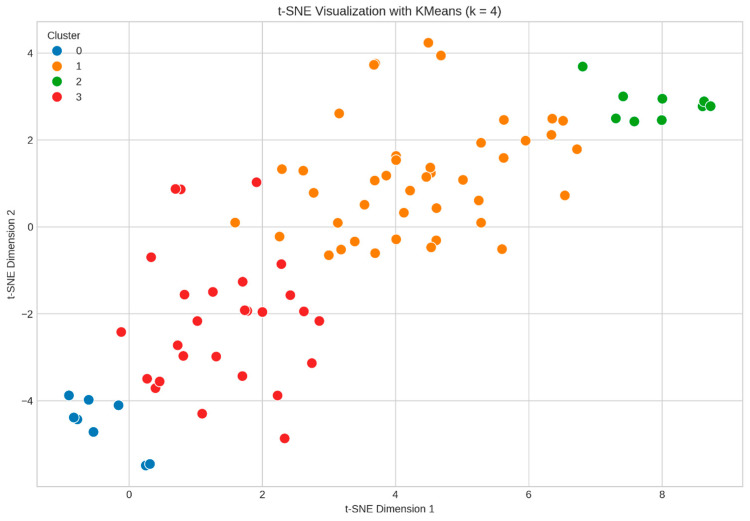
K-means clustering of multi-dimensional patient data visualized via t-SNE (k = 4) (Cluster 0—blue—poor responders; Cluster 1—orange—good responders, intermediate compliance; Cluster 2—green—optimal responders; Cluster 3—red—moderate responders, delayed improvements).

Unsupervised K-means clustering (k = 4) identified four distinct patient subgroups based on clinical and psychosocial variables, visualized using t-distributed stochastic neighbor embedding (t-SNE), as shown in Figure 6:

- Cluster 0 (Poor Responders): 8 patients (9.30%)—the lowest CPAP adherence, the poorest quality-of-life outcomes, and the highest residual AHI.
- Cluster 1 (Good Responders): 42 patients (48.84%)—intermediate compliance with gradual improvement over time.
- Cluster 2 (Optimal Responders): 9 patients (10.47%)—the highest CPAP adherence, the lowest residual AHI, and consistently high WHOQOL scores.
- Cluster 3 (Moderate Responders): 27 patients (31.40%)—delayed but steady improvements in adherence and outcomes.

**Figure 7:**
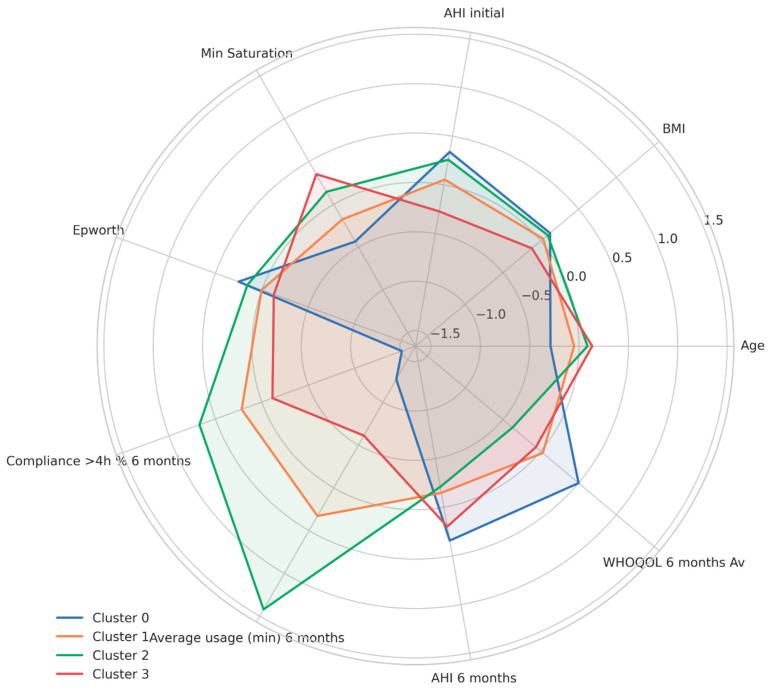
Radar plot of cluster-based patient features.

Standardized cluster profiles based on key clinical, adherence, and quality-of-life variables at six months are presented in Figure 7, highlighting clear multi-dimensional differences among patient subgroups.

#### 3.6.3. Longitudinal Adherence Patterns by Cluster

**Figure 8:**
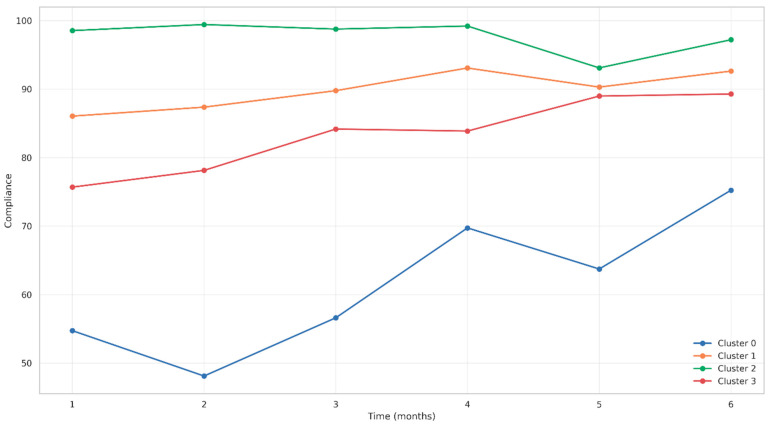
Cluster-based comparison of CPAP adherence trajectories over time.

Distinct adherence patterns were observed across clusters over the six-month period (Figure 8). Cluster 2 maintained consistently high compliance, close to 100% throughout the study. Cluster 1 showed a gradual increase, improving from 86% to over 93%. Cluster 3 demonstrated a steady improvement, rising from approximately 76% to 89%. In contrast, Cluster 0 exhibited the most variable compliance, with values ranging from 55% at the start to 75% by month six.

#### 3.6.4. AHI Evolution by Cluster

**Figure 9:**
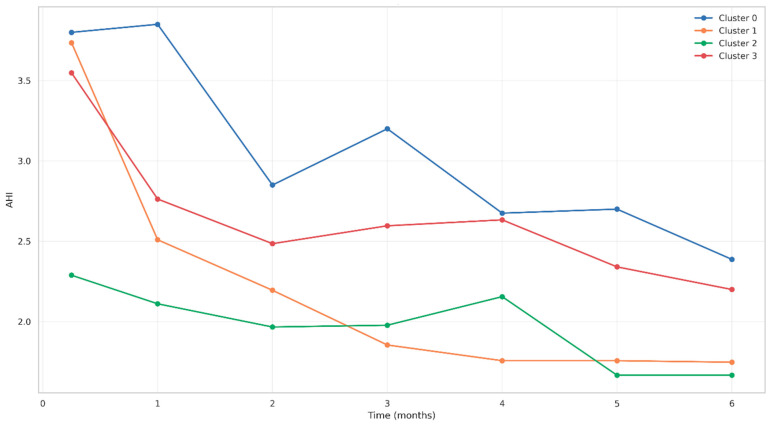
Evolution of AHI by cluster over time.

The evolution of the AHI across clusters showed differential patterns of improvement (Figure 9). Cluster 2 consistently demonstrated the lowest AHI values, stabilizing near 1.7 by month five. Cluster 1 exhibited a similarly positive trajectory with stabilization around 1.8. Cluster 3 showed more gradual decline from approximately 3.6 to 2.2 by month six, while Cluster 0 had persistently elevated and variable AHI values throughout follow-up.

#### 3.6.5. CPAP Usage Patterns by Cluster

**Figure 10:**
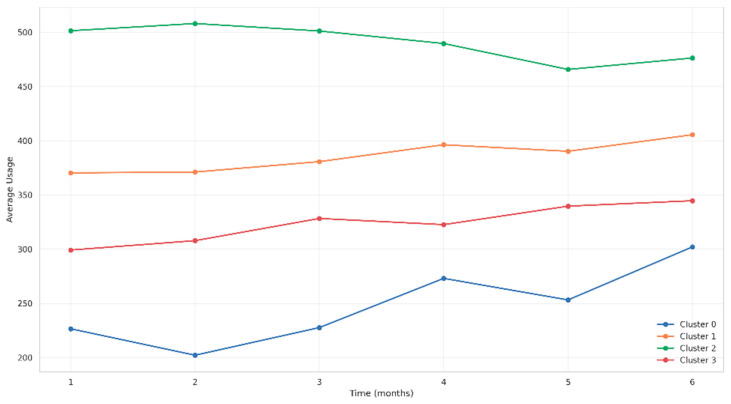
Evolution of average usage by cluster over time.

Average daily CPAP usage patterns differed significantly among clusters (Figure 10). Cluster 2 consistently maintained the highest usage (>470 min/day), while Cluster 1 showed stable upward trends reaching ~405 min/day by month six. Cluster 3 exhibited moderate but steady increases, and Cluster 0, despite starting at the lowest levels (~230 min/day), showed progressive improvement surpassing 300 min/day by the study end.

## 4. Discussion

### 4.1. Clinical Effectiveness of CPAP with Telemonitoring

This study observed that auto-CPAP therapy, when combined with structured telemonitoring via the AirView™ platform and monthly adherence interventions, produces clinically meaningful improvements in patients with OSA. The significant reduction in the AHI from 42.0 ± 21.1 to 1.94 ± 1.31 events/hour aligns with the established literature on CPAP efficacy and suggests effective management of sleep-disordered breathing.

### 4.2. Population Representativeness and Clinical Context

Our study population represents a clinically challenging OSA cohort typical of patients encountered in specialized sleep medicine practice. The predominance of severe OSA (65.1%), substantial obesity burden (79.1% obese), advanced age (mean 60.5 years), and marked baseline functional impairment mirror the demographic and clinical characteristics commonly observed in patients requiring CPAP therapy initiation.

The high prevalence of established adherence risk factors—including severe obesity, advanced age, treatment-naive status, and significant comorbidities—provided appropriate clinical justification for intensive telemonitoring support. This population profile aligns with established predictors of CPAP non-adherence, supporting the external validity of our intervention approach for real-world clinical implementation.

The substantial baseline symptom burden (mean ESS of 17.4, with 61.6% presenting with severe sleepiness) and quality-of-life impairment across all WHOQOL-BREF domains demonstrate the significant functional impact of untreated OSA in this population, emphasizing the clinical importance of successful CPAP therapy initiation and long-term adherence optimization.

### 4.3. Adherence Patterns Through Structured Support

The progressive increase in CPAP adherence observed over six months coincided with structured telemonitoring interventions. The structured approach of providing monthly telephone support to non-compliant patients may represent a contributing factor to the observed adherence patterns. These findings are consistent with previous research by Murase et al., who demonstrated that telemedicine interventions can enhance long-term CPAP adherence. Similarly, Cooper et al. demonstrated, in a quality improvement pilot study, that weekly telehealth interventions during the first month of PAP therapy in rural patients significantly improved adherence rates and reduced daytime sleepiness, as reflected by enhanced Epworth Sleepiness Scale (ESS) scores. These results underscore the value of structured, frequent remote contact—especially in populations with limited access to specialty care—aligning well with the approach used in our cohort [31,32].

#### Human-Centered Care in Telemonitoring

An important design element of our intervention was the use of human healthcare professionals for all patient telephone contacts rather than automated or AI-assisted systems. This decision was informed by the psychological vulnerability of our patient population and growing concerns about AI safety in mental health contexts. The recent literature has highlighted potential risks of AI interactions with individuals experiencing depression or emotional distress, including documented cases of adverse outcomes following AI chatbot interactions [33,34,35,36,37].

Our patients presented with significant baseline psychological impairment, including low self-esteem and severely compromised quality of life, conditions commonly associated with depression and anxiety in OSA populations. The human telephone contacts provided not only technical CPAP support but also crucial psychological check-ins, empathetic listening, and clinical assessment capabilities that current AI systems cannot reliably replicate.

This human-centered approach likely contributed to the positive adherence outcomes observed, as patients received genuine empathy, personalized problem solving, and the reassurance of human connection during a challenging treatment adaptation period. Future telemonitoring protocols should carefully consider the psychological needs of patient populations when designing intervention delivery methods, prioritizing human contact for vulnerable groups despite potential efficiency gains from automation.

### 4.4. Comprehensive Quality-of-Life Improvements

The substantial improvements across all WHOQOL-BREF domains indicate that CPAP therapy with telemonitoring positively influences patients’ overall well-being beyond respiratory parameters. The notable enhancement in self-esteem scores (from 20.1 ± 5.9 to 30.2 ± 5.4) suggests that improved sleep quality translates into better psychological functioning and daytime performance.

These findings align with research by Chavouzis et al., who found that self-esteem improvements are closely related to sleep quality enhancements rather than just objective sleep apnea severity measures [38].

### 4.5. Clinical Phenotyping and Personalized Medicine

The identification of four distinct patient clusters through machine learning analysis reveals the heterogeneity within OSA populations and supports the need for personalized treatment approaches. The clear differences in adherence patterns, clinical outcomes, and quality-of-life improvements between clusters suggest that

1. Optimal responders (Cluster 2—10.47%) may require minimal intervention beyond standard care.
2. Poor responders (Cluster 0—9.30%) need intensive, tailored interventions to improve outcomes.
3. Intermediate responders (Clusters 1 and 3—80.24%) may benefit from targeted support strategies.

This phenotypic approach aligns with the emphasis by Hnatiak et al. on the importance of tailored rehabilitation programs to address individual patient needs [39].

### 4.6. Subjective vs. Objective Outcomes

The strong correlation between subjective improvements (quality of life and self-esteem) and objective measures (AHI reduction) supports the findings of Kang et al., who reported that subjective sleep quality is a primary determinant of quality of life in OSA patients. This emphasizes the importance of addressing both objective sleep parameters and subjective patient experience in OSA management [40].

### 4.7. Critical Evaluation of Psychological Outcome Measures

While our study initially appeared to demonstrate substantial psychological benefits, rigorous post hoc analysis revealed significant methodological concerns that fundamentally challenge these conclusions.

The very large effect size observed for self-esteem improvement (Cohen’s d = 1.78) substantially exceeds typical therapeutic benefits reported in controlled psychological interventions and raised legitimate concerns about measurement validity. The most problematic was the negligible correlation between psychological changes and all objective clinical parameters (highest r = 0.164), indicating that apparent improvements occurred independently of actual treatment effectiveness.

The inverse dose–response relationship—where patients with lower CPAP adherence paradoxically showed greater self-esteem improvements—directly contradicts basic therapeutic principles. Our cluster analysis reinforced these concerns, revealing that patients achieving the best objective clinical outcomes demonstrated smaller psychological improvements than those with relatively poorer clinical responses.

These patterns are entirely consistent with well-documented artifacts in uncontrolled studies.

Hawthorne Effect: The intensive telemonitoring protocol with monthly provider contact likely created strong expectation effects independent of CPAP therapeutic mechanisms. Patients’ awareness of being closely monitored through “advanced” telemedicine technology may have generated optimism and positive self-reporting bias.

Social Desirability Bias: Monthly telephone interactions with healthcare providers created implicit pressure for patients to report improvements, particularly given the time and resources invested in their care.

Response Shift Bias: CPAP treatment initiation may have altered patients’ internal standards for evaluating self-esteem and quality of life, making pre–post comparisons methodologically invalid.

Placebo Response to Technology: The perceived sophistication of the telemonitoring intervention likely generated therapeutic optimism independent of actual sleep apnea treatment effects.

These findings align with meta-analytic evidence showing limited clinically meaningful psychological improvements in general OSA populations treated with CPAP, particularly when not specifically selected for baseline psychological symptoms. Our results underscore the critical importance of controlled study designs when evaluating subjective outcomes in medical interventions.

### 4.8. Gender and Demographic Considerations

The male predominance (72.1%) in our cohort reflects typical OSA demographics. However, previous research by Tasbakan et al. and Kahal et al. suggests that female patients with OSA may experience more pronounced quality-of-life impairments and have specific comorbidity patterns (such as PCOS) that require gender-specific management approaches. Future studies should consider stratified analyses by gender to optimize treatment protocols [41,42].

### 4.9. Study Limitations

Several limitations should be acknowledged.

Primary Methodological Limitation: The single-arm observational design constitutes the study’s most significant limitation, as it prevents definitive attribution of observed improvements to the telemonitoring intervention. Without a control group—such as sham CPAP, waitlist controls, or standard CPAP care without telemonitoring—it is not possible to distinguish the true therapeutic effects from placebo responses, expectation effects, or measurement bias. Moreover, natural adaptation to CPAP therapy, regression to the mean, and unrelated temporal trends may all contribute to the observed outcomes. This design limitation is particularly relevant for subjective measures like self-esteem and quality of life, where causal inference is inherently fragile in the absence of comparator arms. As such, the specific contribution of telemonitoring components remains unquantified.

Psychological Outcome Validity Concerns: Our comprehensive analysis demonstrates that self-reported psychological improvements likely represent measurement artifacts rather than genuine therapeutic benefits. The minimal explained variance by objective clinical measures (3.3%) and inverse dose–response patterns provide compelling evidence of predominant placebo effects rather than treatment-mediated improvements.

Assessment and Reporting Bias: The intensive patient–provider contact inherent in our telemonitoring intervention, combined with unblinded outcome assessment, introduced systematic bias favoring positive self-reports independent of actual clinical benefit.

Selection Bias: Patients willing to participate in intensive telemonitoring protocols may represent a particularly motivated subset with different baseline characteristics and response patterns compared with the general OSA population.

Limited Generalizability: Results are specific to the AirView™ telemonitoring platform and ResMed Auto CPAP devices, potentially limiting external validity to other telemedicine systems or CPAP technologies.

Follow-up Duration: Six-month follow-up may not capture long-term adherence patterns.

### 4.10. Clinical Implications

The results support the integration of telemonitoring systems into routine OSA management, particularly for

- Early identification of non-adherent patients.
- Targeted interventions based on patient phenotypes.
- Comprehensive outcome assessment beyond traditional respiratory parameters.

### 4.11. Future Research Directions

Larger Multicenter Studies: They should be conducted to enhance generalizability and validate clustering findings.

Extended Follow-up: Longitudinal analysis beyond six months should be performed to assess long-term adherence sustainability.

Real-time Interventions: Behavioral feedback mechanisms should be integrated into telemonitoring systems.

Predictive Modeling: Supervised machine learning should be employed to develop personalized adherence prediction models.

Gender-Specific Analyses: Stratified studies should be performed to develop gender-tailored intervention protocols.

## 5. Conclusions

This prospective study observed that auto-CPAP therapy supported by structured telemonitoring via the AirView™ platform and targeted monthly adherence interventions was associated with substantial objective clinical improvements in patients with obstructive sleep apnea. Over six months, patients achieved excellent apnea–hypopnea index reduction (42.0 to 1.9 events/hour), high treatment adherence rates (90.5%), and sustained device usage, confirming the effectiveness of this telemedicine approach for core therapeutic outcomes.

The successful implementation of unsupervised machine learning techniques identified four distinct phenotypic response clusters, demonstrating significant patient heterogeneity in treatment response patterns and supporting the development of personalized management strategies. This data-driven approach to patient phenotyping represents a valuable contribution to precision medicine in sleep disorder management.

However, our comprehensive post hoc analysis revealed critical limitations in the validity of self-reported psychological improvements observed in this uncontrolled study design. The minimal correlation between psychological outcomes and objective clinical measures (R^2^ = 0.033), inverse dose–response patterns, and paradoxical cluster-level relationships provide compelling evidence that apparent self-esteem and quality-of-life improvements reflect placebo effects, response bias, and measurement artifacts rather than genuine therapeutic benefits.

These findings underscore both the substantial effectiveness of telemonitoring for supporting CPAP adherence and achieving clinical treatment goals while simultaneously highlighting the essential need for controlled study designs when evaluating subjective outcomes in medical interventions. The dramatic disconnect between robust objective clinical improvements and artifactual psychological changes demonstrates the importance of methodological rigor in distinguishing genuine therapeutic effects from measurement bias.

This study contributes valuable evidence supporting the clinical implementation of telemonitoring in OSA management while serving as an important methodological case study demonstrating the necessity of controlled comparisons for subjective outcome validation. Future randomized controlled trials with appropriate control groups are required to definitively establish any psychological benefits of OSA treatment with structured telemedicine support.

The code can be viewed and copied at https://colab.research.google.com/drive/1fUYEKXTj9NbfFzYSU0tyCXQTgmnjJAZY?usp=sharing. URL (accessed on 8 July 2025).

## Figures and Tables

**Table 1:** Descriptive characteristics of the study population (n = 86).

Variables	Mean ± SD	Min	Median	Max	n %
Demographics					
Age (years)	60.5 ± 11.9	34	60	89	
Sex—male (%)	72.1%	-	-	-	
Sex—female (%)	27.9%	-	-	-	
Body Mass Index (BMI, kg/m^2^)	35.3 ± 8.0	22.0	33.5	68.0	
BMI categories					
Normal weight (<25 kg/m^2^)	-	-	-	-	1 (1.2)
Overweight (25–29.9 kg/m^2^)	-	-	-	-	17 (19.8)
Obese Class I (30–34.9 kg/m^2^)					27 (31.4)
Obese Class II (35–39.9 kg/m^2^)					19 (22.1)
Obese Class III (≥40 kg/m^2^)					22 (25.6)

**Table 2:** Sleep apnea severity and respiratory parameters.

Variables	Mean ± SD	Min	Median	Max	n %
Moderate OSA (15–29.9 events/hour)	23.1 ± 4.0 *	-	-	-	30 (34.9)
Severe OSA (≥30 events/hour)	52.1 ± 22.4 *	-	-	-	56 (65.1)
Apnea–hypopnea index (AHI)—baseline	42.0 ± 21.1	16.8	33.9	104.5	-
AHI—after 7 days	3.5 ± 4.0	0.2	2.3	22.5	-
AHI—1 month	2.7 ± 2.1	0.3	2.2	11.7	-
AHI—2 months	2.3 ± 1.6	0.3	2.0	9.3	-
AHI—3 months	2.2 ± 1.6	0.2	2.0	9.2	-
AHI—4 months	2.2 ± 1.5	0.3	1.8	7.7	-
AHI—5 months	2.0 ± 1.4	0.2	1.6	6.4	-
AHI—6 months	1.9 ± 1.3	0.1	1.6	5.7	-
Desaturation index	41.3 ± 22.0	9.9	36.3	104.1	-
Minimum O_2_ saturation (%)	70.8 ± 12.0	38	-	87	-
Average O_2_ saturation (%)	91.1 ± 4.0	-	-	-	-

* Mean AHI within severity category.

**Table 3:** CPAP compliance over time.

Variables	Mean ± SD	Min	Median	Max
Compliance after 7 days (%)	75.5 ± 23.9	14.0	81.0	100.0
Compliance >4 h/night—1 month (%)	81.2 ± 17.4	6.0	84.0	100.0
Compliance >4 h/night—2 months (%)	82.1 ± 18.3	16.0	87.0	100.0
Compliance >4 h/night—3 months (%)	85.9 ± 13.8	37.0	90.0	100.0
Compliance >4 h/night—4 months (%)	88.7 ± 12.8	35.0	90.5	100.0
Compliance >4 h/night—5 months (%)	87.7 ± 13.1	37.0	91.0	100.0
Compliance >4 h/night—6 months (%)	90.5 ± 10.1	42.0	91.5	100.0

**Table 4:** Average daily CPAP usage (in minutes).

Variables	Mean ± SD	Min	Median	Max
Average usage—1 month (min/day)	348.4 ± 85.8	60	348.0	538.0
Average usage—2 months (min/day)	349.9 ± 87.2	72	367.0	560.0
Average usage—3 months (min/day)	362.7 ± 73.7	193	365.5	581.0
Average usage—4 months (min/day)	371.5 ± 72.8	184	379.0	560.0
Average usage—5 months (min/day)	369.6 ± 73.2	192	376.0	562.0
Average usage—6 months (min/day)	384.2 ± 65.2	210	374.5	568.0

**Table 5:** Psychological and quality-of-life outcomes at baseline and after six months of CPAP therapy.

Variables	Mean ± SD	Min	Median	Max	n %
Psychological Assessments					
Rosenberg Self-Esteem Scale—baseline	20.1 ± 5.9	10.0	19.0	37.0	-
Rosenberg Self-Esteem Scale—6 months	30.2 ± 5.4	18.0	31.0	39.0	-
Low self-esteem (<15)	-	-	-	-	15 (17.4)
Low-normal self-esteem (15–25)	-	-	-	-	53 (61.6)
Normal self-esteem (>25)	-	-	-	-	18 (20.9)
STOP-BANG score	5.0 ± 1.5	3.0	5.0	8.0	-
Epworth Sleepiness Scale	17.4 ± 3.9	8.0	18.0	24.0	-
WHOQOL-BREF Domains					
Physical health—baseline	36.7 ± 25.4	0	32.0	82.0	-
Psychological health—baseline	32.5 ± 27.0	0	25.0	79.0	-
Social relationships—baseline	20.6 ± 19.1	0	17.0	58.0	-
Environmental health—baseline	36.9 ± 25.4	0	36.0	84.0	-
Physical health—6 months	75.2 ± 21.2	29	82.0	100	-
Psychological health—6 months	71.9 ± 24.1	12	75.0	100	-
Social relationships—6 months	79.8 ± 16.5	33	83.0	100	-
Environmental health—6 months	75.9 ± 21.9	16	82.5	100	-

## Data Availability

The datasets generated during and/or analyzed during the current study are not publicly available due to privacy (GDPR) restrictions. However, anonymized datasets may be available upon reasonable request and with appropriate ethical approval. Requests to access the datasets should be directed to Dr. Ovidiu Fira-Mladinescu (mladinescu@umft.ro).
